# Utilizing spatial statistics to identify cancer hot spots: a surveillance strategy to inform community-engaged outreach efforts

**DOI:** 10.1186/1476-072X-13-39

**Published:** 2014-10-10

**Authors:** Corrine W Ruktanonchai, Deepa K Pindolia, Catherine W Striley, Folakemi T Odedina, Linda B Cottler

**Affiliations:** Department of Epidemiology, University of Florida, 2004 Mowry Road, PO Box 100231, Gainesville, FL 32610-0231 USA; Clinton Health Access Initiative, 383 Dorchester Avenue, Boston, MA 02127 USA; Department Pharmaceutical Outcomes and Policy, University of Florida, 1225 Center Drive, HPNP 3334, PO Box 100496, Gainesville, FL 32610-0496 USA; Department of Radiation Oncology, University of Florida, Health Science Center, PO Box 100385, Gainesville, FL 32610-0385 USA

**Keywords:** Spatial epidemiology, Geographic information systems, Community-engagement research, Community outreach, Health research recruitment

## Abstract

**Background:**

Utilization of spatial statistics and Geographic Information Systems (GIS) technologies remain underrepresented in the community-engagement literature, despite its potential role in informing community outreach efforts and in identifying populations enthusiastic to participate in biomedical and health research. Such techniques are capable not only of examining the epidemiological relationship between the environment and a disease, but can also focus limited resources and strategically inform where on the landscape outreach efforts may be optimized.

**Methods:**

These analyses present several spatial statistical techniques among the HealthStreet population, a community-engaged organization with aims to link underrepresented populations to medical and social care as well as opportunities to participate in University-sponsored research. Local Indicators of Spatial Association (LISA) and Getis-Ord Gi*(d) statistics are utilized to examine where cancer-related “hot spots” exist among minority and non-minority HealthStreet respondents within Alachua County, Florida, United States (US). Interest in research is also reported, by minority status and lifetime history of cancer.

**Results:**

Overall, spatial clustering of cancer was observed to vary by minority status, suggesting disparities may exist among minorities and non-minorities in regards to where cancer is occurring. Specifically, significant hot spots of cancer were observed among non-minorities in more urban areas throughout Alachua County, Florida, US while more rural clusters were observed among minority members, specifically west and southwest of urban city limits.

**Conclusions:**

These results may help focus future outreach efforts to include underrepresented populations in health research, as well as focus preventative and palliative oncological care. Further, global community engaged studies and community outreach efforts outside of the United States may use similar methods to focus limited resources and recruit underrepresented populations into health research.

**Electronic supplementary material:**

The online version of this article (doi:10.1186/1476-072X-13-39) contains supplementary material, which is available to authorized users.

## Introduction

Despite the United States’ (US) National Institutes of Health’s call for inclusion of more broadly representative groups within clinical research [1], racial and ethnic minorities, older populations (aged >65 years), individuals residing in rural areas, and individuals categorized as having lower socio-economic status remain underrepresented in research studies throughout the US [2, 3]. By excluding these populations, national and global research is affected in several ways: 1) risks and benefits of research participation are inequitably distributed across populations [3]; 2) opportunities are missed regarding information particularly relevant to underrepresented populations [3]; 3) external validity or generalizability of research results is reduced, and 4) health-related disparities persist, preventing the US Department of Health and Human Service’s *Healthy People 2020’s* goal to “achieve health equity, eliminate disparities, and improve the health of all groups” [4].

Studies have found enrollment and participation in cancer clinical trials to be particularly low among all patient groups, with underrepresented populations having enrollment rates that are declining [5]. Oftentimes, these groups may be spatially clustered and difficult to reach [6]; it is therefore vital to employ strategic methods to include and retain all populations within research, particularly among marginalized communities such as minority populations, indigenous communities, and populations living in developing countries [7]. The field of community-engagement research works globally to alleviate such underrepresentation, ensuring more broadly applicable findings are generated from epidemiological studies. This approach advocates an ecological perspective, with the rationale that lifestyle, behavior, and illness are all shaped and affected by an individual’s physical and social environment [8]. Further, by addressing such underrepresentation, this approach increases trust within the community and fosters better communication between academic institutions and the community, allowing for future improved health outcomes.

With limited time and resources available, however, it is important to optimize community-engagement outreach efforts in innovative and strategic ways, ensuring the greatest number of community members are informed of potential research opportunities and linked with potential preventative or palliative care, as appropriate. One strategy being utilized internationally with locations throughout the United States and Australia is HealthStreet, a community-engagement model which gathers self-reported head-to-toe conditions over the course of the lifetime and presents a spatially explicit methodology to collect data in real-time. Employing a Community Health Worker (CHW) outreach approach, HealthStreet promotes more rigorous and representative research by engaging individuals within the community in which they live [9]. CHWs travel daily within the community to link residents with a variety of social, legal, and medical resources based upon need, as well as recruit for University-sponsored research studies. By actively approaching and engaging these individuals within the community, HealthStreet enables historically “hidden” or disenfranchised populations to be more effectively reached and referred to programs, services, and research opportunities [9]. Utilization of spatial statistics combined with these community-engaged data collection efforts represents a potential method to guide community-engagement outreach efforts to reach underrepresented populations who may benefit from health promotion activities, as well as focus recruitment efforts for oncological and other clinical trials [10].

The exploratory analyses presented in this paper represent a case study of how spatial statistics can be utilized to inform community-engaged outreach efforts among HealthStreet CHWs. Specifically, we present statistical methodologies utilizing GIS technology to strategically identify cancer-related “hot spots” among minority and non-minority HealthStreet participants residing within Alachua County, Florida, US. The research objectives of this paper include: 1) describing an internationally scalable and spatially pertinent community-engaged strategy, HealthStreet; 2) assessing the demographics and research perceptions of the HealthStreet population to inform future health research recruitment efforts among historically underrepresented populations; and, 3) identifying “hot spots” of cancer within the study area using several spatial statistics, with aims of informing future community outreach efforts to focus preventative knowledge and access to care.

## Methodology

### HealthStreet

In order to monitor the real-time health problems and concerns of underrepresented populations such as ethnic and racial minorities, CHWs travel throughout a variety of community locations, such as parks, retail centers, shelters, laundromats, grocery stores, and more. Specifically, HealthStreet aims to assess medical problems and health concerns from community residents themselves; engage in bi-directional, health-promoting communication with and for the community; link people to medical and social services and opportunities to participate in research, based on their reported needs and concerns; and, increase the community’s trust in the research enterprise through meaningful collaboration [9].

**Figure 1:**
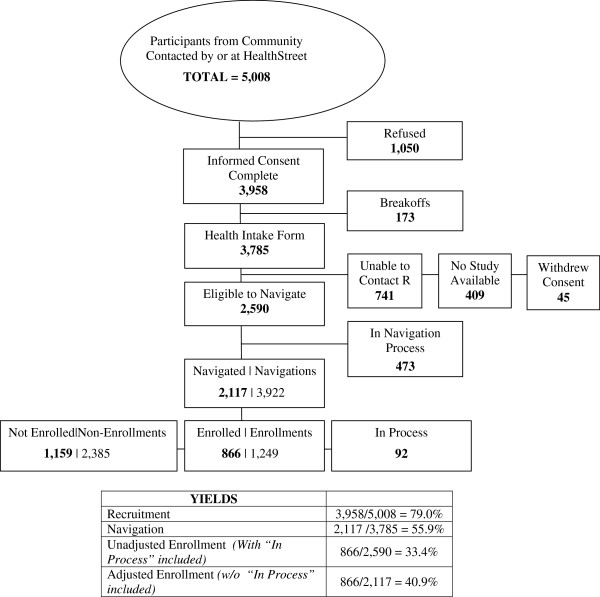
**Participant Flowchart, HealthStreet Gainesville, October 2011 through May 2014.** Numbers in **bold** represent PEOPLE, not events.

Once individuals provide informed consent, CHWs perform a health-based needs assessment, gathering information such as respondent demographics, research attitudes and beliefs, past and present health conditions, health and neighborhood concerns, and prescription and illicit substance use. Respondents are then referred to free or low-cost services within the community, and are “navigated” to IRB-approved studies within the corresponding University community (for this study, University of Florida) based on their health histories. Figure 1 provides information on the number of respondents retained throughout this process, as well as enrollment and recruitment yields for HealthStreet participants seen in Gainesville, Florida, USA from October 2011 through May 2014.

### Institutional Review Board (IRB)

HealthStreet is approved by the University of Florida IRB, with other HealthStreet locations approved by their corresponding IRBs. CHWs administer Informed Consent with each community member and ensure that protected health information (PHI) are confidentially collected and stored. Weekly meetings with HealthStreet staff ensure fidelity to protocols and procedures. Data are collected throughout North Cental Florida and entered using REDCap (Research Electronic Data Capture) software—a secure, web-based data capture tool developed through Vanderbilt University and hosted at the University of Florida [11].

### Data

The HealthStreet sample here was restricted to adults living within Alachua County, Florida, US who completed a Health Intake Assessment between October 2011 and May 2014, resulting in a total sample size of 2,651 community members. To optimize outreach efforts to reach historically underrepresented populations within the US, participants were stratified by minority and non-minority status. Non-minority participants were defined as those reporting non-Hispanic Caucasian race, while minority members were defined as those reporting races of American Indian/Alaskan Native, Asian, African American, Native Hawaiian/Pacific Islander, or Other, as well as those reporting Hispanic/Latino ethnicity. Among this sample, 738 (27.8%) were non-minority, while 1,913 (72.2%) were considered minority. Crude cancer rates are defined in these analyses as number of reported cancer cases divided by total number of participants. A total of 174 (6.6%) participants reported ever having cancer, as measured through the question, “Have you ever been told you had cancer?” on the Health Intake. Reported addresses of residence were geocoded using ArcGIS software, version 10.1 [12], using an address locator created utilizing 2011 Tiger/Line® Shapefiles [13]. Data were projected, and all analyses were performed, using the North American Datum 1983 Universal Transverse Mercator Zone 17 N coordinate system.

While actual address of residence was gathered from respondents, specific household locations were aggregated within a gridded hexagonal surface of dimension 0.050 decimal degrees (n = 2,562 hexagons) to maintain participant confidentiality. Hexagons were generated using Geospatial Modelling Environment (GME) software [14]. A hexagonal surface was used instead of county-level census tracts to 1) reduce potential stigmatization associated with cancer clustering by using a more anonymized polygon, and 2) standardize the number of neighbors adjacent to each polygon for contiguity-based local clustering analyses.

### Analyses

Fisher’s Exact statistics were utilized to examine differences between categorical demographic characteristics and research perceptions among HealthStreet respondents using SAS software, version 9.2 of the SAS System for Windows [14, 15], while t-test statistics were used to examine differences among continuous variables. Copyright © 2009 SAS Institute Inc. SAS and all other SAS Institute Inc. product or service names are registered trademarks or trademarks of SAS Institute Inc., Cary, NC, USA. To measure fair compensation to participate in research, the Wilcoxon Rank Sum test was performed due to non-normal distributions. Kernel Density Estimates (KDE) were calculated to identify regions of the county which might have the highest number of cancer cases among the population, distinct from statistical tests incorporating neighboring areas. This has previously been used in the literature as a quick and easily interpreted method of identifying disease related “hot spots” for focusing community engaged efforts and assessment of services. [16] KDE were calculated using address of residence for both minority and non-minority participants using equal interval bins and a search radius of .015 decimal degrees within ArcGIS version 10.1 software [12]. Multiple bandwidths were examined during analysis; however, default bandwidths were chosen for these analyses, as they represented the most accurate and least biased representation of density based on the input from the team’s knowledge of the data and data collection procedures*.* To present a more concise picture of cancer density, KDE maps are zoomed into central Alachua County to display density estimates for urban regions including the cities of Gainesville and High Springs, Florida, US.

To account for potential rate instability due to a small sample size that reported cancer, smoothed cancer rates (number of observed cancer cases divided by total number of participants) were calculated using Spatial Empirical Bayesian smoothing in GeoDA software [17], using the ‘Calculate Rates’ feature. Variances of smoothed rates versus crude rates were examined, and displayed less variance with smoothed rates. Therefore, smoothed cancer rates were employed for all further spatial analyses, with a contiguity-based spatial matrix with first order queen weights, as opposed to crude cancer rates.

To understand where on the landscape clustering of cancer rates may be occurring, Local Indicators of Spatial Association (LISA) and Getis-Ord Gi*(d) statistics were examined [18, 19]. Using GeoDA software, univariate LISA statistics were performed; Getis-Ord Gi*(d) statistics were performed within ArcGIS software with fixed distance bands of 1 km, 2.5 km, and 5 km, to examine the critical distance at which cancer rates aggregated. More detailed information on Getis-Ord Gi*(d) methods and results is provided in Additional file 1. For LISA statistics, a total of 99,999 randomizations were used with a Type I error rate defined as α = 0.05 to reduce variance instability. By using the maximum number of randomizations, only areas of high, stable significance are reported. Areas produced through this statistic which are defined as ‘high-high’, or “hot spots”, indicate polygons with event rates higher than would be expected by chance, surrounded by similar neighboring polygons with high rates. Conversely, ‘low-low’ areas, or “cold spots”, are areas defined as having lower than expected rates, surrounded by similar areas of low rates. ‘High-low’ and ‘low-high’ polygons are areas of high rates surrounded by lower rates, and vise-versa [19].

## Results

### HealthStreet

Overall, HealthStreet reached a total of 5,008 individuals throughout North Central Florida from October 2011 through May 2014, with 3,785 community members completing a Health Intake Assessment (Figure 1). While nearly 90% of participants expressed interest in participating in research, two-thirds were subsequently eligible to be navigated to a study at the University of Florida. Among the 1,195 who were not eligible, over one-third (n = 409; 34.2%) were ineligible because no study was available to them for which they qualified, as determined by reported health conditions and demographics. Regardless, among the 2,590 eligible participants, 2,117, or over half of those completing a Health Intake, expressed willingness to participate in a specific University of Florida research study for which they were eligible, and were therefore navigated to the corresponding study coordinator. Among these individuals, 866 community members went on to be enrolled 1,249 times into 69 studies, resulting in a final adjusted enrollment yield of 40.9% for HealthStreet participants.

### Alachua County, Florida, USA

Table 1 presents demographics and research perceptions by minority status and self-reported cancer status for HealthStreet participants with reported addresses within Alachua County, Florida, USA limits (n = 2,651). Among those with no history of cancer, minority HealthStreet members (n = 1,913; 74.7%) tended to be younger, never married, with less education than non-minority HealthStreet members. Less differences were observed between minority and non-minority members with a history of cancer, with the exception of education. Among those both with and without a history of cancer, minority members tended to report significantly less education on average than non-minority members.

**Table 1:** **HealthStreet participant demographics and research perceptions (October 2011 - May 2014) by minority status and self-reported lifetime history of cancer, Alachua County, Florida (n = 2,651)**

	No Cancer (n = 2,477)			Cancer (n = 174)				
	***Non-Minority (n = 647)***	***Minority (n = 1,830)***	p-value (a vs. b)	***Non-Minority (n = 91)***	***Minority (n = 83)***	p-value (c vs. d)	p-value (a vs. c)	p-value (b vs. d)
Demographic	a	b		c	d			
	n (%)	n (%)		n (%)	n (%)			
Female	370 (57.2%)	1036 (56.6%)	.922	68 (74.7%)	54 (65.1%)	.187	.003	.214
Mean Age (±SD)	43.0 (±15.1)	39.2 (±15.0)	<.0001	54.7 (±15.6)	51.8 (±15.2)	0.210	<.0001	<.0001
**Marital Status**								
Never Married	268 (41.4%)	1067 (58.3%)	<.0001	26 (28.6%)	27 (32.5%)	.839	.086	<.0001
Married	151 (23.3%)	335 (18.3%)		24 (26.4%)	22 (26.5%)			
Separated/Divorced/Widowed	227 (35.1%)	424 (23.2%)		41 (45.1%)	34 (41.0%)			
Mean Grade Completed (±SD)	13.4 (±2.9)	12.5 (±2.2)	<.0001	14.2 (±3.3)	12.3 (±2.5)	<.0001	.013	.542
Unemployed	395 (61.1%)	1158 (63.3%)	.535	64 (70.3%)	59 (71.1%)	0.913	.236	.412
Veteran	79 (12.2%)	143 (7.8%)	.0009	12 (13.2%)	13 (15.7%)	.671	.920	.043
Food Insecurity	319 (49.3%)	824 (45.0%)	0.141	44 (48.4%)	43 (51.8%)	.825	.303	.063
Medically Uninsured	280 (43.3%)	694 (37.9%)	.040	26 (28.6%)	21 (25.3%)	.733	.016	.050
Seen a Doctor in Past 6 Months	421 (65.1%)	1114 (60.9%)	0.060	76 (83.5%)	71 (85.5%)	.835	.0003	<.0001
**Research Perceptions**								
Ever been in a health research study	150 (23.2%)	279 (15.2%)	<.0001	33 (36.3%)	20 (24.1%)	.010	.027	.105
Interested in being in research study	601 (92.9%)	1647 (90.0%)	.082	87 (95.6%)	81 (97.6%)	.684	.562	.067
Would participate in a study…								
If they were only asked about their health	614 (94.9%)	1694 (92.6%)	.086	88 (96.7%)	74 (89.2%)	.071	.607	.313
If they needed to provide access to their medical records	573 (88.6%)	1541 (84.2%)	.011	81 (89.0%)	72 (86.7%)	.816	.900	.659
If they had to give a blood sample	581 (89.8%)	1503 (82.1%)	<.0001	83 (91.2%)	71 (85.5%)	.342	.852	.593
If they had to take medicine	424 (65.5%)	984 (53.8%)	<.0001	63 (69.2%)	53 (63.9%)	.520	.610	.239
If they had to stay overnight in a hospital	474 (73.3%)	1234 (67.4%)	.014	70 (76.9%)	65 (78.3%)	.712	.526	.018
If they had to use medical equipment	555 (85.8%)	1433 (78.3%)	<.0001	81 (89.0%)	73 (88.0%)	0.827	.516	.098
If they didn’t get paid	532 (82.2%)	1370 (74.9%)	.0003	84 (92.3%)	68 (81.9%)	.049	.029	.053
What they thought was an average fair amount for a study lasting an hour and a half and involving an interview and a blood test	$45.28 (±$73.09)	$87.62 (±$146.60)	<.0001	$40.24 (±$40.30)	$51.15 (±$41.14)	.059	.940	.061

Among both minority and non-minority members, those reporting a lifetime history of cancer (n = 174; 6.6%) tended to be older and reported having seen a doctor within the past 6 months. Among minority members, those with a history of cancer tended to be separated, divorced, or widowed as compared to those with no cancer. While not significant, those with a history of cancer reported participation in a health research study at a rate one and a half times of those who had no history of cancer, regardless of minority status. Further, community members with a history of cancer tended to report higher willingness and enthusiasm to participate in research for less money compared to those not reporting cancer, regardless of minority status.

### Kernel density estimates

**Figure 2:**
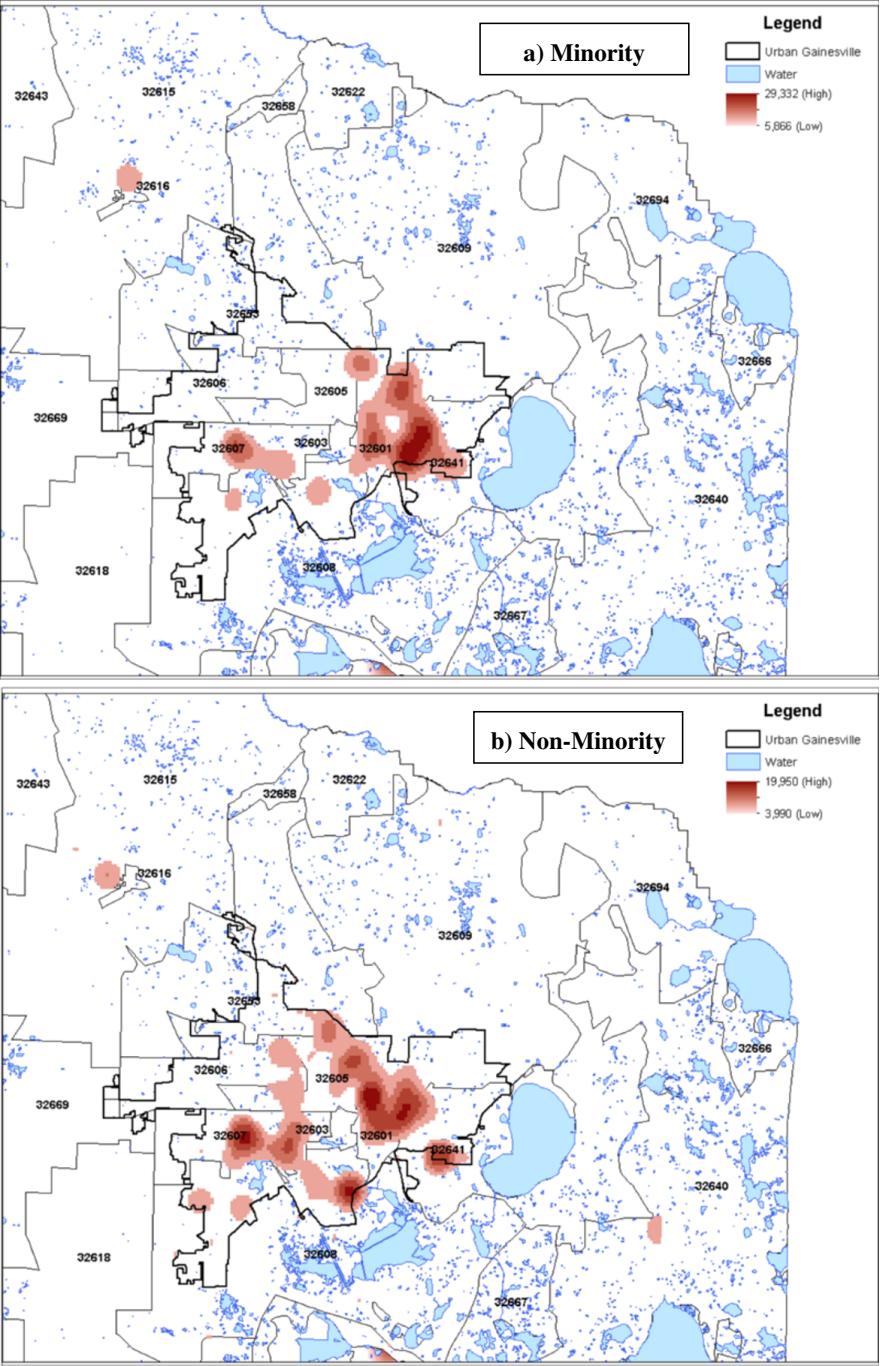
**Kernel Density Estimates (KDE) of self-reported cancer among (a) minority and (b) non-minority HealthStreet respondents, Alachua County, Florida (n = 2,651).** Scale unit in decimal degrees.

Kernel density estimates among minority and non-minority HealthStreet respondents are shown in Figure 2, zoomed in on the Gainesville, Florida urban limits as defined through the US Census Bureau. The highest density of cancer rates is seen predominantly within the urban confines of Gainesville, regardless of minority status. Among urban Gainesville, a higher density of self-reported cancer is seen in the 32641 and 32607 ZIP codes, regardless of whether a respondent was minority or non-minority.

### Local clustering analyses

**Figure 3:**
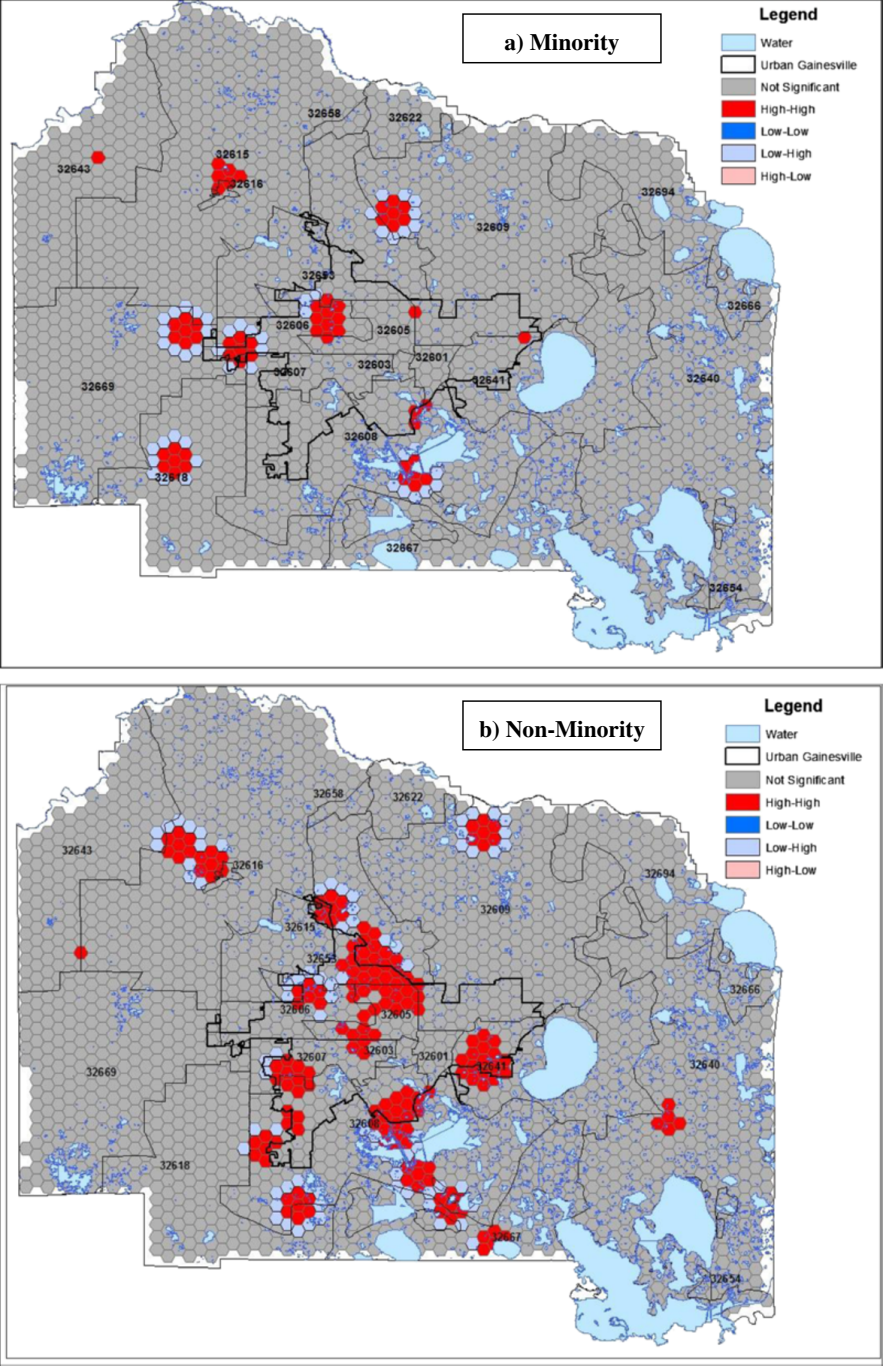
**Local Indications of Spatial Association (LISA) of smoothed cancer rates among (a) minority and (b) non-minority HealthStreet respondents, Alachua County, Florida (n = 2,651).**

Local clustering of self-reported smoothed cancer rates among minority and non-minority HealthStreet respondents as measured through the LISA statistic [18] were examined (Figure 3). The areas on the landscape with significantly high or low rates surrounded by neighboring areas of similarly high or low rates represent areas of high clustering or dispersion. Conversely, areas of significantly high or low rates surrounded by neighbors of significantly opposing rates represent spatial outliers. Among minority residents, high-high clusters were predominantly observed rural areas around Alachua County, Florida, USA, particularly within the west and southwest portions of the county. Among non-minority residents, more clusters were observed in the urban confines of Gainesville, with outlying clusters in the northwestern and southern regions of the county.

Critical distance of case clustering for the Getis-Ord Gi*(d) statistic is shown in Additional file 1, as determined by analyzing distance, *d*, on the scales of 1 KM, 2.5 KM, and 5 KM. Smaller, more localized clusters (as defined by a smaller critical distance) were observed among minority members in rural areas of Alachua County, Florida, USA and northwest Gainesville, while larger, more dispersed clusters were seen in the eastern portions of Gainesville city limits. Among non-minority members, however, clusters tended to be dispersed throughout all of the urban confines of Gainesville, with smaller localized clusters observed in the northern rural areas of the county.

## Discussion and conclusions

Given the vast toolset spatial epidemiologists have at their disposal, including a variety of global and local clustering statistics as well as descriptive spatial statistics, it is important to establish appropriate evidence-based methodologies. These analyses found that local spatial statistics tended to generally agree on where clusters occurred, with regions within Alachua County, Florida, USA consistently showing clustering, regardless of minority status. Figures 3 and A1 (see Additional file 1) show smaller, more localized cancer clusters among minority members within rural areas of the county than those who were non-minority members, with “hot spots” located in the west and southwest rural areas. Such findings may be used not only to direct ongoing and future outreach efforts, but may have policy implications within the area in terms of service assessment and resource allocation. Further, these findings are consistent with previous oncological literature, suggesting minority populations, particularly in rural areas throughout the US have disproportionately high cancer morbidity and mortality. This may potentially be a result of increased barriers to obtaining preventative screenings which can catch lesions before cancer progression [20]. The results of these analyses therefore suggest that if patterns are present on the landscape, such local clustering techniques may have the appropriate resolution to consistently identify them, regardless of test statistic used.

Utilization of these spatial methodologies to identify areas with higher proportions of community members reporting cancer offers a globally relevant, efficient method to focus HealthStreet outreach efforts. With several locations throughout the US and in Australia, a diverse population may be informed of potential opportunities to participate in research and linked to care as a result. Further, previous studies have found disparities in US enrollment rates among historically underrepresented groups, particularly within the oncological realm [5]. Within North Central Florida, however, we found that many community members, especially those with history of cancer, have vastly expressed *more* willingness to participate in research than have reported actually participating (Table 1). These findings are consistent with previous studies utilizing a multi-site, national cohort with information collected from nearly 6,000 respondents throughout the United States [21]. Given these results, there is a discernible need to provide these individuals with opportunities to participate in health research.

Despite this, utilization of spatial statistics remain underrepresented in the community-engagement literature and face unique challenges, such as increased variance and instability of data due to small base rates, confidentiality concerns, and uncertainty as to which spatial statistical technique is most appropriate [22]. These analyses present several techniques which may be utilized by researchers within the global community-engaged field to strategically focus outreach efforts, given limited time and resources. Such efforts not only work to promote and sustain more ethical research practices, but build trust and increased collaboration between researchers and the community, ultimately resulting in improved and sustainable health outcomes.

### Limitations

These analyses acknowledge a variety of limitations: 1) As is common with much oncological literature, sparse data within rural sections of Alachua County, Florida, USA may strongly impact spatial analyses. While Bayesian smoothing may help to alleviate such rate instability, a polygon with little representation may have a biologically implausible rate of self-reported cancer. While the findings from these analyses are consistent with prior literature on the subject, future analyses utilizing HealthStreet data will include increased sample size from a national cohort such as Sentinel Network [21] and will incorporate a Bayesian framework for spatial analyses.

2) The HealthStreet population is a non-random sample, and results therefore may not be generalizable to all populations or countries. However, because HealthStreet aims to over-sample historically underrepresented communities, these results represent an important and traditionally overlooked population.
3) Cancer rates used in these analyses were self-reported and over the course of the lifetime. While it is plausible that most individuals know whether or not they have been diagnosed with cancer, undiagnosed cancer or early stage cancer may persist in the population undetected, thereby increasing the probability of committing a Type II error. Alternatively, cancer rates reported were not necessarily current, suggesting the possibility that cancer “hot spots” identified could be due to past environmental or socio-economic factors. Regardless, these findings help to focus our attention on areas with the greatest need for opportunities to participate in research and increased access to care.
4) Lastly, self-reported rates of cancer were generic to all types of cancer. Different patterns and courses of progression may emerge with varying types of cancer, particularly in regards to gender-specific cancers. Further, clinical utility of results may vary with varying types of cancer. While HealthStreet gathers information on type of cancer the respondent was diagnosed with, these analyses were exploratory in nature, and sought to maximize the number of cases reported. Analyses therefore did not differentiate between types of cancer reported.

### Future directions

Future analyses should work to address the above limitations, particularly in regards to sample size and types of cancer examined. In order to help alleviate the burden of decreased sample size, future analyses may consider focusing efforts within urban regions only; however, important patterns among rural populations may be missed in this scenario. Future analyses should also work to examine predictors associated with reporting a history of cancer, utilizing regression models which take observed spatial autocorrelation into account. As demonstrated by the results of this paper, spatial autocorrelation is an important factor when examining cancer rates. Because observations closer in space tend to be more alike than those farther away [23], it is necessary to account for these patterns and dependence when interpreting or performing statistical analyses, as such data violate assumptions of independence [24]. Finally, future analyses should continue to examine clusters and cluster morphology utilizing a variety of other geospatial techniques, software, polygon shapes, and methodologies.

### Conclusion

The results of these exploratory analyses can be used to provide community-engaged organizations and researchers with an established methodology for examining local clustering for diseases and health conditions, with aims of focusing outreach efforts to increase research participation and linking community residents with valuable medical and social services. With a current enrollment rate at over 40% and rising, HealthStreet is an effective and internationally scalable model which can link historically underrepresented and disparate populations to research which is relevant to their needs. By increasing enrollment of these populations, health research may become more broadly generalizable and health disparities may be reduced, within both research and healthcare settings.

## Electronic supplementary material

Additional file 1: Methods and results for Getis-Ord Gi*(d) analyses. (PDF 985 KB)

## References

[1] National Institutes of Health (2001). NIH Policy and Guidelines on the Inclusion of Women and Minorities as Subjects in Clinical Research—amended October.

[2] Satern WB, Trimble EL, Abrams J, Brawley O, Breen N, Ford L, McCabe M, Kaplan R, Smith M, Ungerleider R, Christian MC (2002). How sociodemographics, presence of oncology specialists, and hospital cancer programs affect accrual to cancer treatment trials. J Clin Oncol.

[3] Ford JG, Howerton MG, Lai GY, Gary TL, Bolen S, Gibbons MC, Tilbur J, Baffi C, Tanpitukpongse TP, Wilson RF, Powe NR, Bass EB (2007). Barriers to recruiting underrepresented populations to cancer clinical trials: a systematic review. Cancer.

[4] US Department of Health and Human Services: *Healthy People 2020*. [http://www.healthypeople.gov] 10.3109/15360288.2015.1037530 26095483

[5] Murthy VH, Krumholz HM, Gross CP (2004). Participation in cancer clinical trials: race-, sex-, and age-based disparities. J Am Med Assoc.

[6] Alvarez RA, Vasquez E, Mayorga CC, Feaster DJ, Mitrani VB (2006). Increasing minority research participation through community organization outreach. West J Nurs Res.

[7] Lavery JV, Tinadana PO, Scott TW, Harrington LC, Ramsey JM, Ytuarte-Nuñez C, James AA (2010). Towards a framework for community engagement in global health research. Trends Parasitol.

[8] Centers for Disease Control and Prevention: *Principles of Community Engagement, Second Edition*. [http://www.atsdr.cdc.gov/communityengagement/pdf/PCE_Report_508_FINAL.pdf]

[9] Cottler LB, Striley CW, O’Leary CC, Ruktanonchai CW, Wilhelm KA, Alving B, Dai K, Chan SHH (2013). Engaging the Community in Research with the HealthStreet Model: National and International Perspectives. Translational Medicine—What, Why, and How: An International Perspective, Volume 3.

[10] Boulous DNK, Ghali RR, Ibrahim EM, Boulos MNK, Abdel MP (2011). An eight-year snapshot of geospatial cancer research (2002–2009): clinico-epidemiological and methodological findings and trends. Med Oncol.

[11] Harris PA, Taylor R, Thielke R, Payne J, Gonzalez N, Conde JG (2009). Research electronic data capture (REDCap) - a metadata-driven methodology and workflow process for providing translational research informatics support. J Biomed Inform.

[12] Environmental Systems Research Institute (2012). ArcGIS Desktop: Release 10.1.

[13] US Census Bureau (2011). TIGER/LINES.

[14] Beyer HL: *Geospatial Modelling Environment (Version 0.7.2.0)*. [http://www.spatialecology.com/gme]

[15] SAS Institute, Inc (2009). SAS version 9.2.

[16] Hart NS, Muhamed S, Das R, Estrella R, Roth J (2013). Neighborhood-level hot spot maps to inform delivery of primary care and allocation of social resources. Perm J.

[17] Anselin L, Ibnu S, Youngihn K (2006). GeoDa: an introduction to spatial data analysis. Geogr Anal.

[18] Ord JK, Getis A (1995). Local spatial autocorrelation statistics: distributional issues and an application. Geogr Anal.

[19] Anselin L (1995). Local indicators of spatial association—LISA. Geogr Anal.

[20] Paskett ED, Tatum C, Rushing J, Michielutte R, Bell R, Foley KL, Bitton M, Dickinson S (2004). Racial differences in knowledge, attitudes, and cancer screening practices among a triracial rural population. Cancer.

[21] Cottler LB, McCloskey DJ, Aguilar-Gaxiola S, Bennett NM, Strelnick H, Dwyer-White M, Collyar DE, Ajinkya S, Seifer SD, O’Leary CC, Striley CW, Evanoff B (2013). Community needs, concerns, and perceptions about Health Research: findings from the clinical and translational science award sentinel network. Am J Public Health.

[22] Goovaerts P, Meliker JR, Jacquez GM (2007). A comparative analysis of aspatial statistics for detecting racial disparities in cancer mortality rates. Int J Health Geo.

[23] Pfeiffer D, Robinson T, Stevenson M, Stevens S, Rogers D, Clements A (2008). Spatial Analysis in Epidemiology.

[24] Legendre P (1993). Spatial autocorrelation: trouble or new paradigm?. Ecology.

