# Supplementary material for: Utilizing spatial statistics to identify cancer hot spots: a surveillance strategy to inform community-engaged outreach efforts
Source: Int J Health Geogr. 2014 Oct 10;13:39. doi: 10.1186/1476-072X-13-39 (PMC4271483; doi:10.1186/1476-072X-13-39)

SUPPLEMENTAL INFORMATION

Methods

An important underlying concept in the geospatial literature is the idea of spatial dependence, in which observations closer in space tend to be more alike than those farther apart [1]. Spatial clustering, or "hot spots", can be defined as the 'spatial aggregation of disease events' or risk factors which are 'unlikely to have occurred by chance [1,2], particularly after known risk factors affecting spatial distribution have been accounted for [3]. Numerous methods exist which examine whether spatial clustering occurs within a given study area, and may be broadly separated into two categories—those producing an overall, single statistic describing *whether* clustering is occurring on the landscape (or global clustering techniques), and those producing statistics which enable detection of *where* clustering is occurring on the landscape (or local clustering techniques) [4].

Both global and local statistics assume a null hypothesis of spatial randomness; however, as previously discussed, real-world data collection frequently does not occur in a randomly selected fashion, with many polygons having little to no representation or data available. In order to adjust for this small base population or high variability in data collection methods, smoothing techniques utilizing Bayesian estimators may be employed, where estimates are interpolated across the study region and "cleaned of noise" based on *a priori* knowledge of the study system [5]. These techniques may help correct for increased variance by calculating a given rate within each polygon that may be averaged against the global or local mean [6]. Both global and local statistics may then be utilized with smoothed prevalence rates, thereby providing more stable estimates, particularly among polygons with a small base population [6].

In order to identify "hot spots", multiple local clustering techniques were utilized, specifically the Getis-Ord Gi*(d) statistic and Local Indicators of Spatial Association (LISA).

*Getis-Ord Gi**

Significant clustering was defined prior to analysis at alpha=0.05, or a resultant Gi* Z-Score of z=1.96, while clustering at increasing distances was defined as occurring when the corresponding z-score also increased. Because this statistic is heavily dependent upon and varies with distance, *d*, it is important to note that both small and large distances *d* may result in the loss of normality [7]; it is therefore critical to perform

multiple G(d) calculations with a variety of distances in order to determine the scale on which aggregation occurs.

In order to visualize clustering at varying distances on the landscape, Gi*(d) z-scores were examined at three distances, 1 km (d_1), 2.5 km (d_2), and 5 km (d_3). Significant clustering was defined on these scales using the following query definitions:

1 km = $Gi^*z(d_1) \geq 1.96$ and $Gi^*z(d_1) > Gi^*z(d_2)$ and $Gi^*z(d_1) > Gi^*z(d_3)$

2.5 km = $Gi^*z(d_2) \geq 1.96$ and $Gi^*z(d_2) > Gi^*z(d_1)$ and $Gi^*z(d_2) > Gi^*z(d_3)$

5 km = $Gi^*z(d_3) \geq 1.96$ and $Gi^*z(d_3) > Gi^*z(d_2)$ and $Gi^*z(d_3) > Gi^*z(d_1)$ and $Gi^*z(d_2) > Gi^*z(d_1)$

Using the above criteria, ‘critical distance’ was defined as the distance at which clustering occurred for any given hexagon using the above queries. This distance could then be represented as a 4-class choropleth map, with classes representing no significance, critical distance defined as 1 km, critical distance defined as 2.5 km, and critical distance defined as 5 km, respectively.

Results

Getis-Ord Gi Critical Distance*

Figure 1 represents local clustering of self-reported cancer rates among minority and non-minority HealthStreet participants as measured by the Getis-Ord Gi*(d) on three distances: a) 1 km, b) 2.5 km, and c) 5 km. Areas of dark red represent significant clustering while areas of dark blue represent significant dispersal. As would be expected, clusters grew in size with increasing distance, d, suggesting the Getis-Ord Gi*(d) is heavily reliant upon distance. Similar to the LISA statistic, clusters among minority participants were observed predominantly in the rural sections of Alachua County, while clusters among non-minority residents were observed in the urban sections of Alachua County.

References

1. Pfeiffer D, Robinson T, Stevenson M, Stevens S, Rogers D, Clements A: *Spatial Analysis in Epidemiology*. Oxford, England: Oxford University Press; 2008.
2. Knox EG: **Detection of disease clusters.** In *Methodology of Enquiries into Disease Clustering*. Edited by Elliott P. London, England: Small Area Health Statistics Unit; 1989:17-20.
3. Wakefield JC, Kelsall JE, Morris SE: **Clustering, cluster detection and spatial variation in risk.** In *Spatial Epidemiology – Methods and Applications.* Edited by Elliott P, Wakefield JC, Best NG, Briggs DJ. Oxford, England: Oxford University Press; 2000:128-152.
4. Aldstadt J: **Spatial Clustering.** In *Handbook of Applied Spatial Analysis: Software Tools, Methods and Applications.* Edited by Fischer MM, Getis A. Berlin/Heidelberg, Germany: Springer-Verlag; 2012:279-300.
5. Berke O: **Exploratory disease mapping: Kriging the spatial risk function from regional count data.** *Int J Health Geo* 2004, **3**:18.
6. Lai PC, So FM, Chan KW: *Spatial Epidemiological Approaches in Disease Mapping and Analysis.* Boca Raton, USA: CRC Press; 2009.
7. Getis A, Ord JK: **The Analysis of Spatial Association by Use of Distance Statistics.** *Geogr Anal* 1992, **24**:189-206.

Figure A1. Getis-Ord Gi*(d) Clustering among Minority and Non-Minority HealthStreet Respondents (n=2,651) at **(a)** 1 KM, **(b)** 2.5 KM, and **(c)** 5 KM.

Minority | **Non-Minority**

a. 1 KM

b. 2.5 KM

c. 5 KM

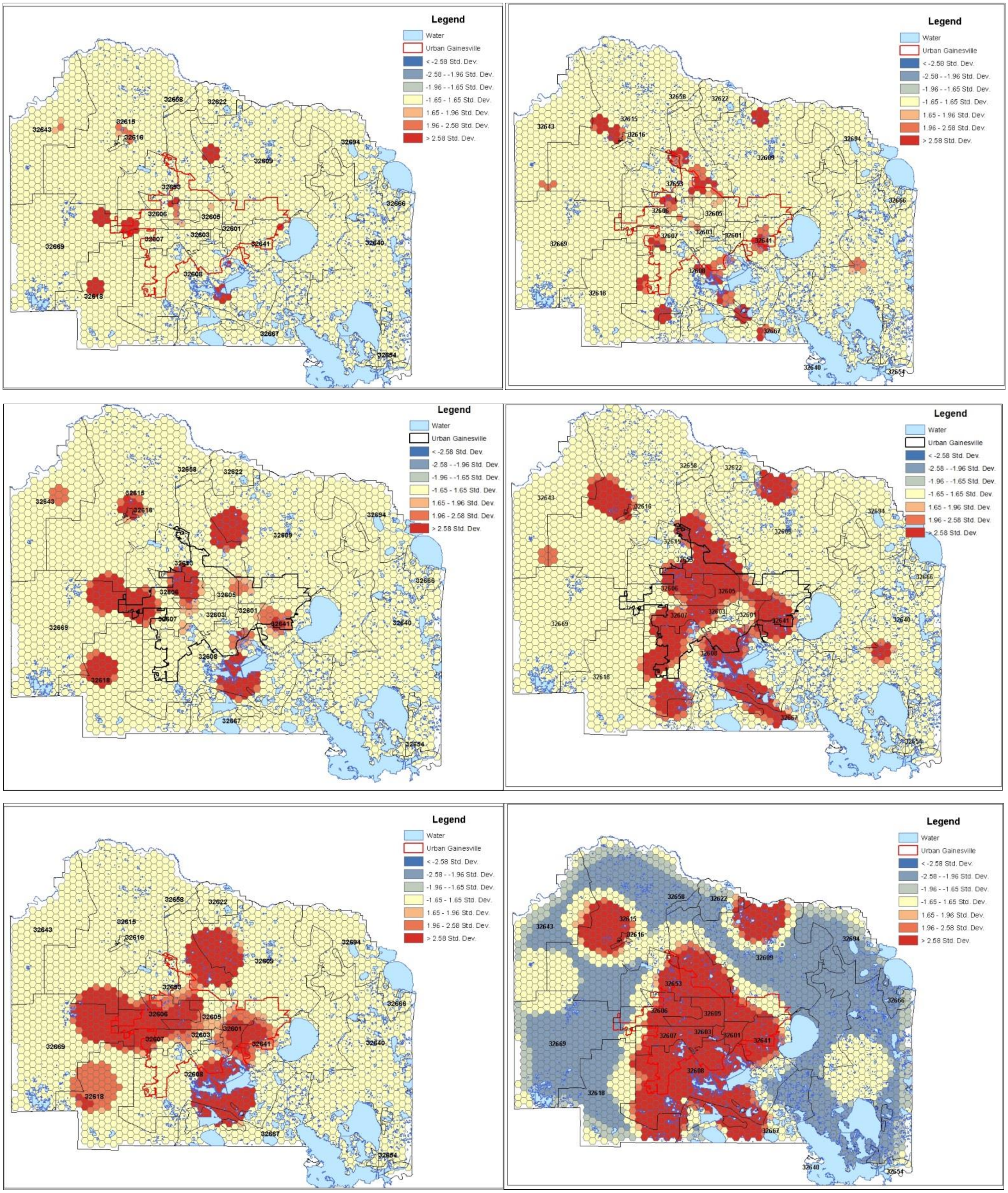

Supplement: Supplementary file 1 — Additional file 1: Methods and results for Getis-Ord Gi*(d) analyses. (PDF 985 KB) [file 12942_2014_611_MOESM1_ESM.pdf]
